# Designing and testing of a health-economic Markov model to assess the cost-effectiveness of treatments for Bipolar disorder: TiBipoMod

**DOI:** 10.3389/fpsyt.2022.1030989

**Published:** 2022-11-10

**Authors:** Anne Kleijburg, Joran Lokkerbol, Eline J. Regeer, Bart Geerling, Silvia M. A. A. Evers, Hans Kroon, Ben Wijnen

**Affiliations:** ^1^Department of Health Services Research, Care and Public Health Research Institute (CAPHRI), Maastricht University, Maastricht, Netherlands; ^2^Centre of Economic Evaluations & Machine Learning, Trimbos Institute, Netherlands Institute of Mental Health and Addiction, Utrecht, Netherlands; ^3^Altrecht Institute for Mental Health Care, Outpatient Clinic for Bipolar Disorder, Utrecht, Netherlands; ^4^Dimence Mental Health Institute, Centre for Bipolar Disorder, SCBS Bipolaire Stoonissen, Deventer, Netherlands; ^5^Department of Psychology, Health and Technology, Centre for eHealth and Wellbeing Research, University of Twente, Enschede, Netherlands; ^6^Department of Social and Behavioural Sciences, Tranzo Scientific Center for Care and Welfare, Tilburg University, Tilburg, Netherlands; ^7^Department of Reintegration and Community Care, Trimbos Institute, Netherlands Institute of Mental Health and Addiction, Utrecht, Netherlands

**Keywords:** economic evaluation, Bipolar disorder, cost-effectiveness, open-source, manic or depressive episode, health economic modeling, Markov model

## Abstract

**Background:**

Bipolar disorder is an often recurrent mood disorder that is associated with a significant economic and health-related burden. Increasing the availability of health-economic evidence may aid in reducing this burden. The aim of this study is to describe the design of an open-source health-economic Markov model for assessing the cost-effectiveness of interventions in the treatment of Bipolar Disorders type I and II, TiBipoMod.

**Methods:**

TiBipoMod is a decision-analytic Markov model that allows for user-defined incorporation of both pharmacological and non-pharmacological interventions for the treatment of BD. TiBipoMod includes the health states remission, depression, (hypo)mania and death. Costs and effects are modeled over a lifetime horizon from a societal and healthcare perspective, and results are presented as the total costs, Quality-Adjusted Life Years (QALY), Life Years (LY), and incremental costs per QALYs and LYs gained.

**Results:**

Functionalities of TiBipoMod are demonstrated by performing a cost-utility analysis of mindfulness-based cognitive therapy (MBCT) compared to the standard of care. Treatment with MBCT resulted in an increase of 0.18 QALYs per patient, and a dominant incremental cost-effectiveness ratio per QALY gained for MBCT at a probability of being cost-effective of 71% when assuming a €50,000 willingness-to-pay threshold.

**Conclusion:**

TiBipoMod can easily be adapted and used to determine the cost-effectiveness of interventions in the treatment in Bipolar Disorder type I and II, and is freely available for academic purposes upon request at the authors.

## Introduction

Bipolar disorder (BD) is an often recurrent mood disorder that is characterized by episodes of depression and (hypo)mania alternated with periods of remission (1). In a largescale pooled analysis from the World Mental Health survey the lifetime prevalence’s for BD type I (BD-I), type II (BD-II), and subthreshold were 0.6, 0.4, and 1.4%, respectively (2). During a depressive episode patients generally experience strong feelings of sadness and hopelessness, a loss of pleasure and interests in normal activities, and even suicidal thoughts. During episodes of mania patients may experience a strong increase in energy, feelings of excessive euphoria or agitation and a decreased ability to sleep and control impulsive behavior. Depending on the severity and duration of manic episodes BD can be classified according to four types, type I, type II, cyclothymia and unspecified or subthreshold BD. BD-I describes patients experiencing manic episodes with a duration of at least one week and a severity that significantly limits their functioning, and is more likely to result in psychosis or require admission. BD-II describes patients experiencing hypomanic episodes with a duration of at least four days where manic symptoms are less severe and functioning is affected but not limited. With cyclothymia patients experience milder forms of manic and depressive mood episodes, and patients with symptoms indistinctive of the other types are classified as unspecified or subthreshold (1, 3).

Treatment options for BD generally consist of both pharmacological and non-pharmacological interventions that aim to prevent the relapse of manic and depressive episodes, decrease the severity of manic and depressive symptoms, and improve inter-episodic functioning (4–6). As BD is most commonly diagnosed during early adulthood, its lifelong and highly variable nature often requires long-term treatment, whilst introducing significant detriments to the coping individual’s quality of life and productivity (7, 8). Consequently, BD is associated with both substantial healthcare costs and productivity losses, incurred by patients as well as caregivers, introducing a significant economic burden on society (7, 9–12).

When aiming to create an efficient and sustainable healthcare system, policy-makers require not only information on the effectiveness of interventions but also their relative value for money, as this guides decisions ultimately impacting a finite healthcare budget (13). Such decisions can for example encompass whether or not to implement new (treatment) strategies in practice, to adopt certain treatments over others in new clinical guidelines, or to reimburse treatments by health insurers. Economic evaluations can provide decision-makers with such information by determining the relative efficiency and costs (or cost-effectiveness) of new interventions when compared to current interventions. When the evidence needed to perform an economic evaluation is not available from a single source, decision-analytic models allow combining data from various sources and its extrapolation over a sufficiently long time horizon while explicitly taking into account uncertainty (14, 15).

Given the significant economic and health-related burden of BD, as well as the wide variety of interventions that exist for the treatment of BD, we believe that a better understanding of their relative cost-effectiveness may aid to reduce this burden. Therefore, the aim of this study is to present and describe a flexible decision-analytic model, Trimbos institute’s BipoMod (TiBipoMod), that can be used to examine the long-term cost-effectiveness of user-defined pharmacological and non-pharmacological interventions in the treatment of adults with Bipolar Disorder type I (BD-I) and type II (BD-II). The model will be made available for all researchers with interest upon request. Similarly, easily adaptable decision-analytic models aiming to increase the availability of cost-effectiveness evidence for treatment and prevention are already available for psychosis and depression (16, 17). To provide complete transparency toward its potential users, this paper describes (1) the process of developing a conceptual model, (2) the final structure of the model and its assumptions, (3) the parameters used by the model, and 4) a case study to illustrate the use and results generated by the model. Overall, the model’s details described here may aid its users in the process of adapting the model and its parameters to match the context and research question at hand.

## Materials and methods

### Model development

As the aim of this study is to create a flexible decision-analytic model to examine the long-term cost-effectiveness for treatment of Bipolar Disorder, TiBipoMod was developed as an easy-to-use Microsoft Excel-based Markov cohort model. In a Markov cohort model a cohort of patients is modeled over a predefined time period during which they transition between the various included health states, accumulating costs and health effects associated with each health state given the treatment condition over time (15).

The model was developed in line with the guidelines of the Professional Society for Pharmacoeconomics and Outcomes Research (ISPOR) for conceptualizing a model (18). First, the research problems to be answered with this model were formalized, providing the foundations for the conceptual model. To conceptualize the model structure, a scoping literature review on the disease progression of BD and existing cost-effectiveness studies was performed, after which its final structure was validated by an expert panel (see below). The expert panel for this study consisted of two healthcare professionals in the treatment of BD in the Netherlands who were consulted throughout the development process to validate assumptions and parameter values.

After finalizing the model structure, health state parameters and model assumptions were formulated using available treatment guidelines, national databases, published literature and expert opinions. This iterative process finally resulted in the following PICOT for TiBipoMod:

• Population: Adults with the diagnosis of Bipolar Disorder, type I and type II, as defined by the 2013 DSM-V^1^ (1).
• Intervention: User-defined interventions are modeled in addition to the reference treatment(s), and compared to the standard of care (SOC) alone. In order to model an intervention, users are required to insert the relative risks of experiencing a manic and depressive episode given the intervention of interest, and its associated costs. The model is able to compare two interventions simultaneously using separate Markov traces, and present its outcomes. Interventions may be pharmacological and non-pharmacological.
• Comparator: The modeled comparator is the SOC, which by default has been parameterized based on clinical treatment guidelines and expert opinion. The SOC may be easily adapted to a user-defined SOC by adjusting parameter values. By default, the comparative scenario includes commonly prescribed pharmacotherapy and psychotherapy, outpatient mental specialist care, community treatment, and episode crisis care.
• Outcome: Costs per quality-adjusted life year (QALY) gained.
• Time Horizon: Given the lifelong nature of BD, costs and health effects are modeled over a lifetime horizon, but also a 5-year horizon when shorter horizons are preferred. The model uses a cycle-length of three months.

To provide in the varying demands of guidelines for health-economic evaluation both a healthcare and societal perspective can be applied, and future costs and effects are discountable by user-defined rates (by default: 4 and 1.5%, respectively) (19). A half-cycle correction is applied to account for the fact that transitions between states may occur at any time during the cycle (20).

For deciding on a willingness-to-pay (WTP) threshold to be applied, country-specific guidelines for economic evaluation can be used or, when absent, the WHO recommends using a threshold of three times the national GDP (21). For example, in the Netherlands the guidelines for Disease Burden in economic evaluations provides WTP thresholds based on disability weights (22). According to the Global Burden of Disease 2013 study BD disability weights are estimated at 0.40 and 0.49 for depressive and manic episodes, respectively, resulting in a recommended WTP threshold of €50,000 in the Netherlands (23). When using a GDP-based threshold this would result in a WTP of €147,300 (2021 GDP in the Netherlands: €49,100).

### Model conceptualization

The first step in the development of TiBipoMod was to explore disease progression of BD and the conceptualization of BD in published health economic models by performing a scoping literature review. In this process ten model-based health-economic evaluations for the treatment of BD were identified (Supplementary Material I). In this and in clinical literature six potential health states became apparent that were to be considered for model inclusion; depression, mania, hypomania, rapid cycling, remission/euthymia, and death. Whereas mania, depression and remission were found in previous economic evaluations, hypomania and rapid cycling were not (6, 24–29). Reasons in the literature for the exclusion of hypomania as a separate health state are the lack of evidence surrounding parameters for hypomania, and that the burden imposed on the patient by hypomania is considered less severe than during a depressive or manic episode of BD-I. As for rapid cycling, clinical guidelines stated that depending on the polarity of the episode this is treated as either a manic or depressive episode. Finally, patients with BD experience an elevated risk of suicide and higher mortality rates due to comorbidity and poorer lifestyle choices throughout their life course, contributing to an overall reduction in life-expectancy, supporting the inclusion of death as health state (30–32).

Based on the considerations above, our conceptual model aimed to include the health states depression, (hypo)mania, remission, and death. From the existing models identified during the literature review the schematic model structure published by Ekman et al. (33) best matched these health states and was considered to best fit the Markov modeling approach of this study. In the study of Ekman et al. (33), a discrete event simulation was used to simulate the occurrence of four health states to determine the cost-effectiveness of quetiapine in patients with acute bipolar depression and maintenance treatment (33). The possibility of treatment discontinuation in this model described by Ekman et al. is not included for the current purpose.

As a second step, an expert panel of healthcare professionals was consulted to validate the conceptual model based on Ekman et al. The panel confirmed the structure of the initial conceptual model, however, as the initial model only included a transition from depression to mania, the panel collectively recommended the inclusion of the transition from mania to depression, which may occur in response to the excitatory processes of mania (34, 35). In addition to this, the panel was consulted on the differences between manic and hypomanic episodes including its implications for treatment. The experts stated that despite similarities, important differences are often observed in the time spent in the mood episodes, quality of life, and experienced during episodes.

**FIGURE 1:**
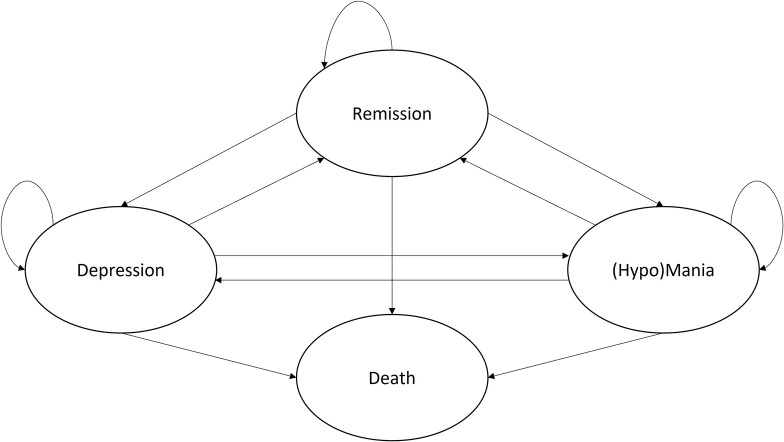
Final conceptual Markov model for the treatment of Bipolar Disorder. Adapted version of the model published by Ekman et al. (33).

Given that the aim of the current model is to represent both BD-I and BD-II, the Markov model was built with two separate Markov traces for each type, estimating costs and effects for both subpopulations, which can then be combined into a single weighted ICER using the proportion in prevalence. The final model structure therefore includes the health states depression, (hypo)mania (i.e., mania for BD-I and hypomania for BD-II), remission, and death (Figure 1).

### Model parameters

#### Mood state epidemiology and transition probabilities

To populate our conceptual model with health state transition probabilities, available literature on the epidemiological characteristics of BD and its longitudinal disease course was reviewed and compared. In this process, multiple studies were identified that report on the differences in long-term symptomatic status of BD-I and BD-II, the time spent in the various mood states, and recurrence rates (36–42). When looking at studies that present the percentages of time spent in various mood states, large variations can be observed in their findings (see discussion) (36, 39–41). Here, comparison and drawing conclusions is challenged by the highly heterogenous study designs and definitions of mood states. As such, a single study was selected to inform prevalence rates as our main guidance in selecting and verifying the modeled epidemiology. Based on the assessment frequency of reported symptoms, the study by Kupka et al. (36) was chosen, which describes the largest naturalistic cohort of patients (n: BD-I = 405, BD-II = 102), where patient’s daily self-reported symptoms were assessed weekly to monthly for one year by their physicians, and translated to DSM-IV mood episodes (36). The following paragraphs describe studies (or study arms) which have been selected to inform model transition probabilities. In those studies all patients are provided with some form of pharmacological treatment typical to the respective mood episode studied, meaning the model does not simulate untreated disease progression, but rather progression given commonly prescribed or naturalistic pharmacotherapy.

First, the probabilities of relapsing from remission to both depression and mania were informed by the literature review and meta-analysis of Vazquez et al. (42) (BD-I: 96% of patients), combined with the reported time spent in depression/mania ratio by Kupka et al. (36) (BD-I: 81% of patients). Vazquez et al. (42) report an annual recurrence rate for any mood relapse while treated with active medications of 21.9% based on 15 RCTs, which could be translated to the quarterly transition probability of 5.99% (42). To correct for differences seen in the time spent in depressive and manic mood episodes, and to match the prevalence of mood episodes seen in BD-I and BD-II epidemiology, days spent in depression and mania (excluding days with mild/subsyndromal symptoms) reported by Kupka et al. (36) were used to construct a depression/mania ratio (36). This resulted in depression/mania ratio for BD-I of 4.7 and BD-II of 10.7, which combined with the probability of recurrence by Vazquez et al. (42) resulted in the final probabilities for relapsing to mania or depression presented in Table 1. The probability of remaining in remission was found by subtracting the probabilities of leaving the health state.

**TABLE 1:** TiBipoMod state transition probabilities per (three-month) cycle and health state utilities.

	BD-I	BD-II	Distribution	References
Proportion BD-type population	0.600	0.400	Beta	(2)
**Time spent mood states**				
*Ratio Depression/Mania*	4.7	10.7		(36)
*% Time in Depression*	0.744	0.792	Beta	(36)
*% Time in Mania*	0.256	0.208	Beta	(36)
**Health state transitions - SOC**				
*Remission to Remission*	0.880	0.880	Dirichlet	
*Remission to Depression*	0.094	0.117	Dirichlet	(42)
*Remission to Mania*	0.026	0.003	Dirichlet	(42)
*Depression to Depression*	0.561	0.561	Dirichlet	(41)
*Depression to Remission*	0.365	0.365	Dirichlet	
*Depression to Mania*	0.074	0.074	Dirichlet	(33)
*Mania to Mania*	0.298	0.048	Dirichlet	(41)
*Mania to Depression*	0.074	0.074	Dirichlet	(33)
*Mania to Remission*	0.628	0.878	Dirichlet	
**Mortality**				
*RR Premature death*	2.060	2.060	Lognormal	(44)
*RR Suicide*	9.660	9.660	Lognormal	(44)
**Intervention effect (see case study below)**				
*RR Mania*	1.320	1.320	Lognormal	(55)
*RR Depression*	0.810	0.810	Lognormal	(55)
**Utilities**				
*Remission*	0.800	0.800	Beta	(52)
*Depression*	0.290	0.290	Beta	(52)
*Mania*	0.540	0.800	Beta	(52)

Second, to determine the probabilities of remaining in a mood episode, time-to-recovery estimates provided by Solomon et al. (41) were used. This study was performed using data of an observational study where patients of predominantly BD-I patients (*n* = 219) who were not controlled for any somatic treatment received. They report 50% of patients remaining in a major depressive episode after 15 weeks, 25% of patients remaining in a manic episode after 15 weeks, and 25% of patients remaining in a hypomanic episode after 6 weeks, with a median duration for a mood episode of 13 weeks (41). Using R statistical software, exponential regression equations were applied to the reported number of weeks per quantile for patients to recover from each mood episode type to estimate the transition probabilities per model cycle (13 weeks) (43). This results in a probability of remaining in a depressive, manic and hypomanic episode after 13 weeks of 56.1, 29.8, and 4.8%, respectively.

Then, to inform the probabilities of transitioning between mood states, Weibull distributed parameters reported by Ekman et al. (33) (BD-I: 66% of patients) were combined with the relative risks for receiving pharmacotherapy with mood stabilizers and olanzapine. Given these conditions, the probability of transitioning from depression to mania was 7.4% (33). Despite being recognized frequently in clinical practice by the expert panel, little evidence was found on the transition probability of mania to depression. In consultation with the expert panel, the probability of this transition was set equal to the transition of depression to mania (7.4%). The probabilities of transitioning from mania to remission and depression to remission were found by subtracting the probabilities of leaving the health states.

Finally, transitions to death were based on general mortality statistics in the Netherlands as reported by Statistics Netherlands. To account for the increased risk of suicide and death by comorbidities associated with BD relative mortality rate ratios (MRR) from Westman et al. (44) were applied to the general mortality. For comorbidities and lifestyle effects of BD a MRR of 2.06 was applied independent of the health state, and for suicide an additional MRR of 9.65 was applied to the depressive state only, as suicide occurs less frequently during mania or remission (44, 45).

#### Validating modeled epidemiology

**FIGURE 2:**
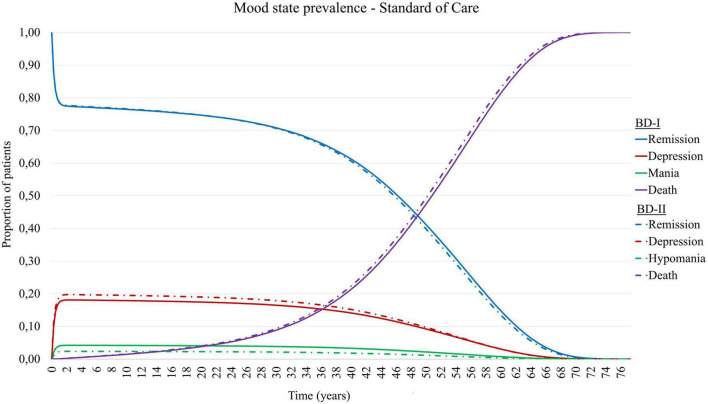
Mood state prevalence of simulated patient cohort with Bipolar Disorder type I (BD-I) and type II (BD-II) in TiBipoMod, consisting of ∼ 4% of live patients in mania, ∼ 18% in depression, and ∼78% in remission.

Combining the above mentioned transition probabilities in the Markov chain resulted in the modeled epidemiology presented in Figure 2. Here, from the patients not transitioned to death, around 78% of patients are in remission, 18% are experiencing a depressive episode, and 4% a manic episode.

To validate the modeled epidemiology with the empirical data by Kupka et al. (36), a comparison was made between the time spent in the various mood states. To this extent, distinctions were made between the severity of mood episodes, as the degree to which functional impairment occurs is an important factor in the increasing need of health services and reduced productivity. As such, we assigned time spent with mild or subsyndromal symptoms to the remission states, and only included time spent with moderate to severe symptoms to the respective health states. Based on the prevalences reported by Kupka et al. this assumption would translate into a guiding estimate of patients spending 74.0-76.2% of time in remission/euthymia, 4.6-7.5% in (hypo)mania and mood cycling, and 18.4-19.1% of time in depression. It is important to note that this distribution was used as a guidance during the process of informing state transition parameters. However, given the variation seen in the available evidence we chose not to calibrate parameters to match these guiding estimates exactly.

Based on the above mentioned heterogeneity and uncertainty (also discussed later), the modeled prevalence estimates were considered in agreement with the guiding estimates reported by Kupka et al. (36).

#### Defining the standard of care

Similar to the heterogeneity in clinical presentation of patients suffering from BD, the amount of health services that patients use is also highly heterogenous (3, 46). In addition to this, a wide variety of treatment options are available for the treatment of BD, including various forms of pharmacotherapy, psychological therapy, community-based treatments, and inpatient treatments. As a result, defining the “standard of care” for the treatment of BD is challenging. Given that this current model aims to serve as a health-economic tool that is easily adaptable by its users to match their needs, by default the model is designed as such that the SOC during each health state included all most commonly provided treatment options identified in the treatment guidelines and by expert opinion (3, 46–48). This resulted in the identification of five major care components that are generally included in treatment regimens for BD and are included in the model. These components are:

1. Pharmacotherapy: For patients with BD pharmacotherapy most commonly consists of either monotherapy or polypharmacy with mood stabilizers, antipsychotics, anticonvulsants or antidepressants, depending on the health state and patient-specific preferences.
2. Outpatient mental specialist care: Routine treatment and monitoring by, e.g., psychiatrists and a mental health nurse (practitioner) is recommended to promote relapse prevention, stimulate self-management, and adjust treatment during episodes.
3. Psychotherapy: Consensus exists on the importance of psychotherapy programs for patients and relatives to stimulate successful long-term management and relapse prevention. Commonly recommended outpatient psychotherapy programs are psycho-education, cognitive behavioural therapy (CGT) and interpersonal and social rhythm therapy (IPSRT).
4. Community-based care: For patients with a more severe form of BD, a form of community-based treatment may be indicated where multidisciplinary teams provide continuous, flexible and outreaching treatment and monitoring. Examples of such service models are collaborative care models, (flexible) Assertive Community Treatment models, and crisis models such as Crisis Resolution Teams or Intensive Home-based Treatment.
5. Inpatient care: During severe mood episodes patients may also be admitted to a psychiatric hospital. Depending on the local availability of alternative crisis treatments, the incidence and duration of hospitalization may vary.

Based on these individual components, the SOC for each of the model health states is assumed to consist of the treatment options presented in Table 2. Here, pharmacotherapy and outpatient specialist care is included for both the remission and mood episode states. Despite commonly being provided during periods of remission, in consultation with the expert panel the majority of psychotherapy sessions has been assigned to the mood episode health states to account for the fact that patients often participate in these upon remitting from an episode. Inpatient care is included only for the mood episode states.

**TABLE 2:** Treatment components of the standard of care per health state included in TiBipoMod.

	Patients assigned to treatment intensity (%)			
		
	Remission	Depression	Mania	References
**Outpatient low intensity treatment** ○ *Pharmacotherapy* ○ *Outpatient mental specialist care*	85%	90%	30%	(45, 53), expert opinion)
**Outpatient high intensity treatment** ○ *Pharmacotherapy* ○ *Outpatient mental specialist care* ○ *Community-based treatment* ○ *Psychotherapy/education*	15%	7%	40%	(45, 53), expert opinion)
**Inpatient care** ○ *Pharmacotherapy* ○ *Outpatient mental specialist care* ○ *Hospital admission*	NA	3%	30%	(45, 53), expert opinion)

To account for the fact that not all patients may need or want to make use of these treatment components during a depressive or manic episode, use of care can be weighted by means of percentages based on three categories for treatment intensity; “outpatient low intensity,” “outpatient high intensity,” and “inpatient care.” For example, patients with a less severe mood episode may receive additional treatment with low intensity, patients with more severe episodes may receive additional treatment with high intensity, and only patients with very severe episodes may be admitted. By default, the category “outpatient low intensity” constitutes of outpatient mental specialist care and community-based treatment with increased frequency as compared to remission care, and “outpatient high intensity” constitutes of outpatient mental specialist care, a psychotherapy program and community-based treatment. Pharmacotherapy is included for all patients. Both the assigned percentage weights and components of the treatment categories can be altered to match the local context of the user.

#### Valuation of cost components

TiBipoMod offers analysis from both a healthcare perspective as well as a societal perspective, including productivity costs and patient and family costs. From the healthcare perspective, direct medical costs consist of pharmaceutical costs, costs relating to outpatient specialist care, psychotherapy, community-based treatment and inpatient care. Indirect medical costs included in the model are the periodic costs for drug-induced renal failure testing and medical costs for unrelated diseases during other and the last year of life, calculated using the tool Practical Application to Include Disease Costs (PAID) (49, 50). Productivity loss estimates associated with BD for absenteeism and presenteeism are included in the model based on literature estimates (9). Costs included in the model for the patient and family are limited to the hours spent by caregivers on informal care (7, 51). All cost components are comprised of individual units for resource use and unit costs. Costs related to the intervention are included by a separate parameter, applied only to the intervention arm. Also, adjustable parameters are included allowing to adjust the number of cycles with which intervention costs and effects are experienced. By default, TiBipoMod will be populated with resource use and unit cost inputs based on the Dutch context (section 3.1), however, as all inputs can be adjusted, we encourage users to adjust accordingly to match their local context.

#### Quality of life

Quality of life experienced during each health state was described using utility scores published by Revicki et al. Utilities are based on both inpatient and outpatient treated patients suffering from BD type I (*n* = 96) and measured using the standard gamble (SG) method (52). Health state utility scores for patients suffering from BD type II were not found in the literature. However, based on the differences in clinical presentation of mood episodes seen with BD type I and type II, differences may be expected in quality of life experienced and thus utility scores. For example, differences in clinical presentation are especially significant for mania and hypomania, where in general hypomania is shorter in duration but, most importantly, not associated with severe functional impairment (1, 3).Therefore, to include QoL estimates in our model better representable for both BD-I and BD-II, assumptions were made based on its clinical presentation and considering the model cycle time of 90 days. As such, we assumed QoL during a hypomanic episode for the patients with BD type II to be equal to the quality of life during remission (0.80), rather than that of mania (0.54), as measured by Revicki et al. Health state utilities for remission and depression (0.29) are assumed equal for BD type I and type II, as presented in Table 1.

### Model outputs

Model outputs of TiBipoMod are expressed in costs, life years (LYs) and QALYs for both the intervention(s) and comparator (discounted and undiscounted). Outcomes are compared using the incremental cost-effectiveness ratio (ICER). The ICER is calculated as followed: (Costs intervention - Costs control)/(QALYs intervention - QALYs control). Here, the ICER represents the incremental costs per QALY gained.

### Probabilistic sensitivity analysis

To assess the uncertainty surrounding the parameters included in the model probabilistic sensitivity analyses (PSA) can be performed. Probability distributions are assigned to each parameter in the model based on its characteristics, e.g., beta distributions for utilities with a value between 0 and 1, the skewed gamma distribution for costs, and Dirichlet distributions for transition probabilities that sum up to 1. For parameters of which its value was to remain between predefined bounds (e.g., in case of treatment guidelines stating a minimum and maximum amount of treatment sessions) a beta-PERT distribution was applied. Subsequently, the PSA can be run 5,000 times, each time drawing a random value from the distribution for each parameter. As incremental costs and effects are simulated 5,000 times they can be plotted in an incremental cost-effectiveness (CE) plane, with the incremental QALYs on the x-axis and the incremental costs on the y-axis, illustrating its uncertainty. In addition to this, a cost-effectiveness acceptability curve (CEAC) is constructed illustrating the likelihood of the intervention being considered cost-effective given a series of willingness-to-pay (WTP) thresholds (15).

### Validation

To validate the final model, both internal and external validations have been performed. First of all, for external validation of conceptual ideas and input parameters the expert opinion panel played a key factor. In addition to this the Assessment of the Validation Status of Health-Economic decision models (AdViSHE) tool was used, a 13-item questionnaire assessing four typologies; conceptual validation, data validation, computerized model validation and operational validation (53). For internal validation the black box test TECH-VER checklist was applied, ensuring technical verification, completeness and consistency (54). The results of both tools are presented in Supplementary Material II.

## Case study: Mindfulness based cognitive therapy in the Dutch context

To apply TiBipoMod to a real-world example, the effectiveness of a mindfulness-based cognitive therapy (MBCT) intervention, as described in the results of a randomized controlled trial performed, was combined with the costs of providing the intervention. The MBCT intervention aims to reduce the chance of relapse, and to reduce the severity of depressive symptoms during an episode. This effect was studied in the RCT by Perich et al. (55), where the intervention group received MBCT and the SOC, and the control group only received the SOC. The RCT’s primary outcome was the 12-month recurrence rates of depressive and (hypo)manic episodes. Despite not being significantly different, 59% of the participants in the MBCT group had suffered a (hypo)manic episode in the past year and also 59% a depressive episode, while in the SOC group 48% of the participants had a (hypo)manic episode and 68% a depressive episode (55). These annual recurrence percentages were calibrated to quarterly recurrence rates and used to determine the relative risks for a mood episode. This resulted in relative risks of 0.81 and 1.32 for transitioning to depression and (hypo)mania when treated with MBCT, respectively. This effect was modeled to persist for 4 cycles (separate parameter).

### Valuation of unit costs

Cost parameters for the SOC in this case-study were determined using a bottom-up costing approach. Direct medical costs incurred to each patient by the use of included treatment options were determined using treatment guidelines (56–58), expert panel estimates, national cost databases (59, 60), and reference prices published in the Dutch manual for cost research (19, 51). For example, costs related to specialized mental healthcare are based on estimates for hours spent on consultations provided by the expert panel, and combined with the hourly reference physician rates. Productivity costs are included in the model up until the Dutch retirement age of 67, were informed by the literature (9). Costs of informal care for the patient and family are valued at the Dutch reference price for unpaid work (7).

Costs relating to the MBCT intervention were based on its 8 sessions in groups of 8 to 12 people offered by two mental health nurse practitioners. This resulted in average additional costs of €291 per person per quarterly cycle for MBCT, which was modeled for a single cycle. The relative risks for (hypo)manic and depressive episodes and its intervention costs were added to the model to determine interventional transition probabilities and costs for MBCT + SOC. All costs were expressed in 2021 Euros by indexing unit cost prices with the consumer price index when necessary (61).

### Sensitivity and scenario analyses

To provide insight in the impact of changes in modeled epidemiology of BD-I and BD-II, two one-way sensitivity analyses were performed with alternative ratios for time spent in depression/mania. For these scenarios the studies of Joffe et al. (40) and Judd et al. (37, 38) were used which found significantly higher ratios for BD-I = 6 and BD-II = 14, and BD-I = 3.6 and BD-II = 38.7, respectively.

### Results

Finally, the cost-effectiveness of MBCT + SOC compared to the SOC alone was determined from a healthcare and a societal perspective. From a societal perspective, MBCT + SOC resulted in an average per-patient increase of 0.017-0.019 QALYs and a decrease in costs of €339- €674 depending on the simulated BD subtype, resulting in a dominant ICER per QALY gained. All outcomes for modeled scenarios are presented in Table 3.

**TABLE 3:** Costs included in the model per patient per (three-month) cycle for each health state.

	Remission	Depression	Mania	Sources
Drugs	€ 39	€ 53	€ 53	(3, 60)
Medical services	€ 161	€ 786	€ 804	(51, 57)
Psychological treatment	€ 271	€ 126	€ 722	(51, 56, 57)
Home-based treatment	€ 131	€ 153	€ 874	(51, 57)
Indirect medical costs	€ 40	€ 40	€ 40	(49)
Productivity losses	€ 443	€ 3,637	€ 2,182	(9, 51)
Patient *and* family costs	-	€ 4,996	€ 2,997	(7, 51)
Admission costs	-	€ 617	€ 6,166	(51, 57)
Intervention costs	€ 291	-	-	(51, 55)

**FIGURE 3:**
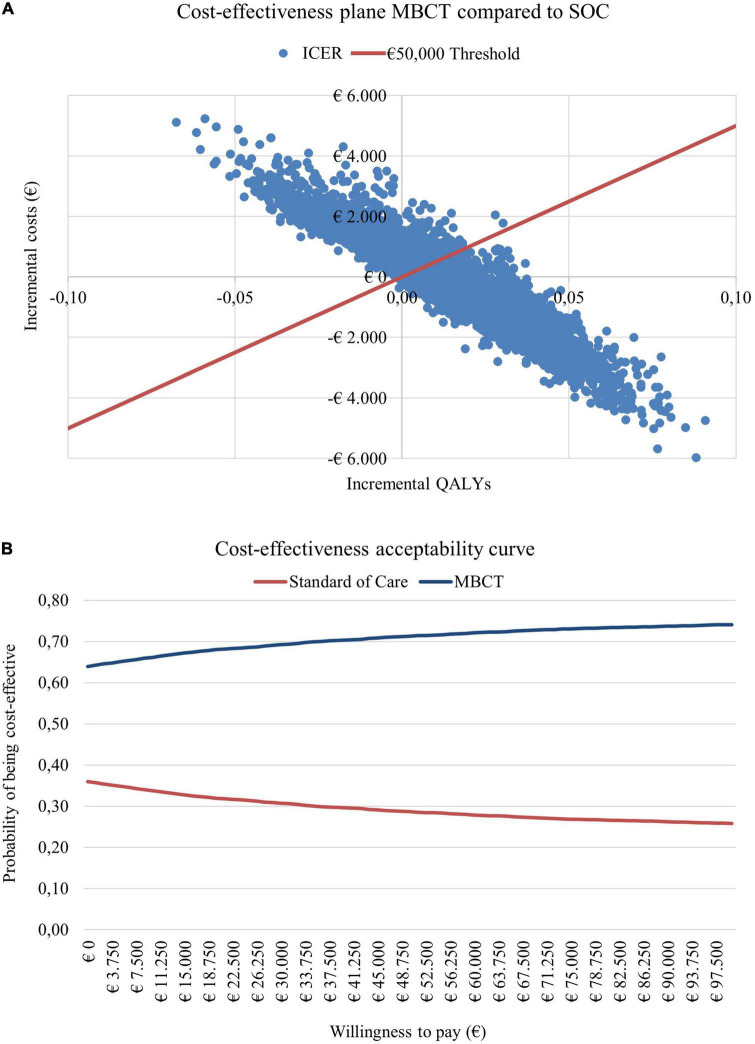
**(A)** Cost-effectiveness plane and **(B)** cost-effectiveness acceptability curve for adding mindfulness-based cognitive therapy (MBCT) to the standard of care (SOC) compared to the SOC alone.

When running sensitivity analyses this resulted in the cost-effectiveness plane and CEAC presented in Figures 3A,B. When considering a WTP threshold of €50,000, there was a 71% probability that MBCT + SOC is cost-effective.

## Discussion

We have presented TiBipoMod, a Markov model that is able to evaluate the (long-term) cost-effectiveness of both pharmacological and non-pharmacological interventions for patients suffering from both BD-I and BD-II. When provided with the necessary input parameters describing the intervention and local context (e.g., relative risks for a depressive and manic episode, intervention costs, expected duration of effect, unit costs), our model is able to present outcomes from a healthcare and societal perspective, a 5-year and lifetime time horizon, and includes various built-in parameters to adjust for the heterogeneity of BD, e.g., in terms of quality of life, functional impairment, healthcare resource use, and differences in epidemiology between BD-I and BD-II. In addition to this, because the model is Excel-based, its use does not require advanced health-economic modeling skills and the model is easily adjustable in its functionalities. The model was developed in line with (inter)national clinical treatment guidelines, available literature on its epidemiology, treatment, intervention effects and costs, and in consultation with Dutch healthcare professionals in the treatment of BD. Additionally, these professionals provided important input in the validation process of our input sources and model assumptions, supplemented by the AdViSHE and TECH-VER validation tools.

To illustrate the outcomes generated by this model a case study was performed assessing the cost-effectiveness of MBCT + SOC compared to the SOC, which found that MBCT + SOC is dominant over the SOC alone when considering a societal perspective, but is associated with an ICER of €15,993 - €28,987 per QALY gained from a healthcare perspective. This difference illustrates the impact of including societal costs such as productivity loss and caregiver costs, and their relevance for inclusion when evaluating interventions for BD.

### Strengths and limitations

Strengths of our model are related to its potential to aid in the generation of cost-effectiveness evidence that is more easily comparable across intervention (compared to outcomes derived from different economic models), while also based on methods that are fully transparent. Though cost-effectiveness of both pharmacological and non-pharmacological interventions in the treatment of BD have been studied and systematically reviewed, common conclusions were drawn that 1) the number of available studies was relatively low and 2) the methods applied were heterogeneous, creating a need for more robust and better comparable (long-term) evidence to inform policy decisions (4–6, 62). In addition to that, increasing interest emerges toward health-economic models that are open source, i.e., available to anyone who wishes to access it (63–65). Important arguments for this have been its potential for increasing knowledge-sharing, efficiency, consistency and, perhaps most importantly, transparency and credibility of evidence generation in cost-effectiveness research thereby reducing uncertainties. Here, transparency is achieved by providing full access and insight to all methods and assumptions made throughout the model (66, 67). In addition to that, TiBipoMod includes additional background information sheets in the Excel model to ensure full disclosure on all sources used and subsequent assumptions made.

The development of the model should be seen in light of some limitations that are important to acknowledge when considering to adopt our model. First, TiBipoMod is constructed as a Markov cohort model, a model type that is widely used in estimating cost-effectiveness resulting from its relative simplicity, transparency and useability whilst often maintaining sufficient accuracy depending on its application (68). However, limitations of Markov models that have been frequently identified in the literature are its lack of memory (i.e., the subsequent health state only depends on the present health state, and not the sequence of preceding states), fixed cycle length and state-transition probabilities, and its limited ability to model complex diseases better represented by a larger numbers of health states (68, 69). For example, the wide variation in duration of mood episodes is not well represented by the fixed blocks of time, and little distinction can be made in the severity of the mood episodes of the respective episodes when represented by a single health state (70–72). When interested in capturing time- or patient-specific effects, one should consider modeling approaches that allow for greater complexity and detail such as discrete-event simulation (DES) models. However, considering the limitations of a DES model, being that its complexity requires advanced modeling skills, resources, and the fact that it is more data heavy, we felt it did not align with our aim of creating an easily-adaptable model, opting for a Markov model with additional parameters attempting to correct for the heterogeneous nature of BD presentation and treatment (73).

A second potential limitation of our model relates to uncertainty following from the epidemiological parameters included in our model. First of all, our transition probabilities have been derived from multiple sources where the study populations existed of varying patient population characteristics, such as the proportion of patients included with a BD-I and BD-II diagnosis (ranging from 66% to 96% BD-I). Multiple studies report on the long-term symptomatic status and time spent in various mood states by patients suffering from BD-I and BD-II (36–41). Although comparability of studies is complicated by several factors, such as the use of different rating scales [i.e., the National Institute of Mental Health Life Chart Methodology (NIMH-LCM) or the Longitudinal Interval Follow-up Evaluation (LIFE) system (74, 75)] discrepancies present, stressing the importance of acknowledging the uncertainty underlying the epidemiology. For example, despite comparable findings on the amount of time spent in remitting phases (44%-54%) of BD-I and BD-II, some studies report on significant differences in time spent in depressive and (hypo)manic episodes (36, 39, 40). However, when looking at reported ratios for time spent in depression/mania per BD subtype, Kupka et al. (36) find relatively comparable ratios for BD-I and BD-II with 2.9 and 3.8 including mild symptoms (when excluding mild symptoms this becomes BD-I = 4.7 and BD-II = 10.7), respectively, and no differences in episode frequency which suggest similar tendencies in mood switching and symptomatic status. When comparing this to the depression/mania ratios found by Joffe et al. (40) (BD-I = 6 and BD-II = 14), and even more so to those found by Judd et al. (37, 38) (BD-I = 3.6 and BD-II = 38.7), these suggest significant differences in clinical course between BD-I and BD-II. Although these discrepancies can be partially explained by differences in mood state definition, study design and patient assessment frequency, favoring the outcomes by Kupka et al. (36), significant uncertainty surrounding the true clinical trajectories of BD subtypes remains. It is therefore important to emphasize that differences in modeled epidemiology for BD-I and BD-II should be subjected to sensitivity analyses, which is also why this feature has been implemented in TiBipoMod.

A third limitation of our model stems from a lack of available evidence to inform model parameters, e.g., for QoL, resource use, health-state transitions and societal losses, either in general or specifically for BD-II when only available for BD-I. For example, the available health state specific SG utilities published by Revicki et al. (52) were measured in BD-I patients only, requiring additional assumptions (52). Collectively, this lack of evidence for QoL, and the subsequent assumptions made introduce additional uncertainty, stressing the need for further research (i.e., especially in BD-II).

A fourth limitation that stems from this lack of evidence also relates to the studies used to inform transition probabilities in this model. Current transition probabilities are based on RCTs or observational studies in which (most) patients have received pharmacological treatment, which treatment(s) exactly, however, is not clear for each study. Therefore, our model simulates interventions that have been added to some form of best practice treatments, including pharmacotherapy, rather than untreated disease progression. As a result, the relative risk for experiencing a mood episode given the intervention considered for evaluation should, ideally, be measured in patients that receive some form of baseline pharmacotherapy.

Fifth, simplifying BD to a model with only four health states is a strong simplification of the true population heterogeneity. In general, BD is characterized by its strongly heterogeneous mood swings, fluctuating somewhere between severe depression and extreme manic states, alternated with periods of remission. Even within the categorization of BD in type I and II or unspecified/subthreshold, the severity of mood episodes may vary per patient and per episode independent of the specific BD diagnosis (3, 46). Similarly, transitions between mood episodes, i.e., mania to depression or depression to mania, are frequently observed but often do not occur consecutively and may be separated by weeks to months of remission (35). However, given this Markov model is population-based it aims to describe the average probability for an event to occur and costs associated, rather than individual sequences of events.

A sixth limitation that stems from this heterogeneity is the wide availability of treatment options available to patients suffering from BD, and a lack of evidence regarding the use of these various options, as well as non-compliance to treatment over time which is currently not included in the model. As such, our model was limited to a selection of treatment options identified by (inter)national guidelines and expert opinion. With regards to the Dutch context, concordance with treatment guidelines assessed in the outpatient setting was found to be high (48). Moreover, as the main source for validating transition probabilities was based on empirical data stemming from the Dutch clinical setting, it is reasonable to assume that the modeled treatments (i.e., as part of SOC) are in line with the interventions provided in the Dutch study.

A seventh limitation concerns the generalizability of TiBipoMod’s current model parameters, structure and assumptions across countries, for example in terms of locally available treatment options and the organization of care nationally. Currently, included treatment components (pharmacotherapy, psychotherapy, and community-based treatment etc.) are based on clinical guidelines published by the National Institute for Health and Care Excellence (NICE) in the United Kingdom and the Dutch National Health Care Institute, therefore likely better representing countries with similar health systems. In addition to that, by default the model is informed with healthcare resource use and unit costs representative of the Dutch context. Also, the model currently does not provide a detailed overview of the various accumulated costs carried across providers, which may be relevant for countries with a multiple payer system. Overall, depending on country-specific contexts, some future users may have to perform more model adaptations, or have limited information available to inform necessary parameters.

A final limitation of this model is that there remains room for further model development and implementation of novel concepts in health economic modeling. Examples of such novel concepts are the use of the expected value of (partial) perfect information (EV(P)PI), the value of hope, the inclusion of a broader societal perspective (i.e., costs related to public health, criminal justice, education, housing, or the environment), or alternative quality of life measures such as the Capabilities Approach, which contrasts the use of utilities in mental health by focusing on an individual’s subjective wellbeing (76, 77). The use of EV(P)PI could, for example, provide insight in the expected costs of the decision uncertainty surrounding model input parameters, such as the transition probabilities. Outcomes of this analysis may identify if additional research is worthwhile, and what consequences could be when adopting the wrong treatment strategy (78, 79).

## Conclusion

We presented TiBipoMod, a Markov model that is able to evaluate the long-term cost-effectiveness of pharmacological and non-pharmacological interventions in the treatment of adults with BD-I and BD-II from a healthcare and societal perspective. Overall, TiBipoMod aims to support researchers in adding conclusive knowledge to the limited health-economic evidence of treatments of BD in the clinical setting, supporting policy makers to make decisions considering the costs and effects of BD treatment. Moreover, TiBipoMod is freely available for academic purposes upon request from the authors. To support the development of this and other health-economic models for BD, future research should focus on increasing the availability of evidence to inform its parameters, and reduce related uncertainty for both BD-I and BD-II.

## Data availability statement

The original contributions presented in this study are included in the article/Supplementary material, further inquiries can be directed to the corresponding author.

## Author contributions

JL and BW: conception and project design. BW, AK, and JL: model development. AK and BW: writing of the manuscript. AK, BW, SE, HK, JL, BG, and ER: critical appraisal of the manuscript. All authors read and approved the manuscript.

## Footnotes

1 As little differences are observed in the classification of bipolar disorder between the DSM-IV and V, studies using the DSM-IV to inform model parameters were also included.
