# Supplementary material for: Designing and testing of a health-economic Markov model to assess the cost-effectiveness of treatments for Bipolar disorder: TiBipoMod
Source: Front Psychiatry. 2022 Nov 10;13:1030989. doi: 10.3389/fpsyt.2022.1030989 (PMC9684337; doi:10.3389/fpsyt.2022.1030989)
Supplement: Supplementary file 1 [file Data_Sheet_1.PDF]

Supplementary Material

Designing and testing of a health-economic Markov model to assess the cost-effectiveness of treatments for Bipolar Disorder: TiBipoMod

Supplementary I: Results of a preliminary scoping review

Overview of previous model-based economic evaluations regarding the treatment of BD

**Authors**	**Modeling technique**	**Health states**	**Time horizon**
(Calvert, Burch et al. 2006)	Markov	Depressed, Manic, Euthymic	18 months
(Cheema, Frangou et al. 2013)	Markov	Euthymic, Manic, Depressive	1 year
(Chisholm, Van Ommeren et al. 2005)	Markov	Manic Depressive, Euthymic	Lifetime (10 year treatment)
(Ekman, Lindgren et al. 2012)	DES	Manic, Depressed, Remission, Death	5 years
(Fajutrao, Paulsson et al. 2009)	Markov	Euthymia, Depression, Mania	24 months
(Klok, Al Hadithy et al. 2007)	DES	Hospitalization simulation	12 weeks
(McKendrick, Cerri et al. 2007)	Markov	Mania, depression, stable	1 year
(Soares-Weiser 2007)	Markov	Manic, Depression, Stable	5 year

(Woodward, Tafesse et al. 2009)	Markov	Depression, Mania, Euthymia, Death	2 year
(Woodward, Tafesse et al. 2010)	Markov	Depression, Mania, Euthymia, Death	2 year

*DES: discrete event simulation.

Supplementary II: Model validation

Assessment of the Validation Status of Health-Economic decision models (AdViSHE)

Part A: Validation of the conceptual model

**Face validity testing (conceptual model):** Have experts been asked to judge the appropriateness of the conceptual model?
- Two experts were consulted throughout the development of this model. The first expert is psychiatrist and senior researcher Dr. E. Regeer from the TOP-GGZ treatment centre Altrecht GGZ, Bipolair, Utrecht, the Netherlands. The second expert is the experienced nurse practitioner and researcher B. Geerling, currently Head of the department for Bipolar Disorders and Psychiatry and Pregnancy at Dimence, a network organization for specialized psychiatric care.<br><br>- Both experts have longstanding experience in the treatment of and research in bipolar disorders.<br><br>- The experts largely agree with the conceptual model, however, do stress that some assumptions or generalisations made throughout the model may differ from clinical practice. Some examples of this are the lack of nuances in direct transitions between mood episodes (between mania and depression), which in practice may be separated by weeks of remission, and the use of treatments during periods of remission rather than manic or depressive episodes, as is the case for psychotherapy or psychoeducation. Despite these treatments being provided more commonly during remission (and sometimes depression but not mania), a larger portion of the costs was assigned to these treatments during the episodes as these treatments are more commonly provided after relapses, and this is difficult to simulate using a Markov model. Their concerns have also been discussed in the discussion section of the manuscript.

**Cross validation testing (conceptual model)**: Has this model been compared to other conceptual models found in the literature or clinical textbooks?
- Prior to involving the experts to test the face validity of the model, a thorough search of previous literature has provided a preliminary model. Previous decision analytic modeling economic evaluations with regards to BD have been compared to grasp the idea of a conceptual model for the current study. Considerations regarding these conceptual models have been discussed with the experts, and improvements were made based on their advice.

Part B: Input data validation

**Face validity testing (input data):** Have experts been asked to judge the appropriateness of the input data?
- The expert panel introduced in Part A was also involved in judging the input data. A first attempt of the assessment of input parameters has been done using national guidelines and previous studies. These parameters have been discussed with the experts to check whether they match reality. Some of the parameters which have been included for health care use in the Dutch context, informing the case study, were not available from literature. The experts gave their estimations on these parameters based on their clinical experience.

**Model fit testing:** When input parameters are based on regression models, have statistical tests been performed?
- The input parameters were not based on regression models, therefore, testing them statistically is not applicable.

Part C: Validation of the computerized model

**External review:** Has the computerized model been examined by modelling experts?
- The model has been reviewed by two co-authors of this study, Dr. Ben Wijnen and Dr. Joran Lokkerbol. Both are experienced health economic modellers who have been involved in many projects at the Centre for Economic Evaluation and Machine Learning at the Trimbos institute. They are also project designers and authors of two

previously published similar open source models for psychosis (PsyMod) and depression (DepMod).

- Additionally, the model and a concept of the manuscript were assessed by an independent, experienced reviewer at the Low Lands Health Economic Study Group conference in May 2022. This conference provides opportunity to researchers to seek in-depth discussion of work-in-progress projects by experienced researchers in the field of health economics that are matched by the conference committee based on their expertise.

- The discussion and feedback on the preliminary manuscript and model presented at the conference can be requested from the authors upon interest. No unresolved issues currently remain.

Extreme value testing: Has the model ben run for specific, extreme sets of parameter values in order to detect any coding errors.

- The model has been run for specific and extreme sets of parameters. Variations in individual parameters and the effect on the results have been reported in the deterministic sensitivity analysis section of the report. Added to that, the internal validity of the model and its input has been tested using the black-box test of TECH-VER.

Testing of traces: Have patients been tracked through the model to determine whether its logic is correct?

- The Markov trace is visible in the model. This way the movement of the cohort can be followed during the entire time-horizon. No odd course has been detected.

Unit testing: have individual sub-modules of the computerized model been tested?

- Yes, individual sub-modules have been tested.

Part D: Operational validation

**Face validity testing (model outcomes):** have experts been asked to judge the appropriateness of the model outcomes?
- The experts (from part A and B) have been consulted to check the appropriateness of the first outcomes of the model. In the case study MBCT reduces the probability of having a depressive relapse, and increases the probability of a manic relapse. According to the model MBCT gains QALYs and Lys, which is expected as the QoL and probability of death are greater during depression. Total incremental costs for MBCT are lower than for CAU when looking from a societal perspective (due to productivity losses), and higher when looking from a healthcare perspective. Treatment costs are higher because the probability of a manic episode is larger, which is more costly episode compared a depressive episode.

**Cross validation testing (model outcomes):** Have the model outcomes been compared to the outcomes of other models that address similar problems?
- The results and intermediate results were compared with epidemiological data on BD. The transitions after one year were compared with the one-year recurrence rates available in literature. Furthermore, it was difficult to cross-validate costs as this is rather country-specific and for the Dutch situation cost of psychotherapy were not found in previous health economic modelling literature.

**Validation against outcomes using alternative input data**: Have the model outcomes been compared to the outcomes obtained when using alternative input data?
- Yes. Multiple scenarios have been tested using alternative epidemiological parameters. Outcomes of these scenarios indicated that conclusions remain consistent.

**Validation against empirical data:** Have the model outcomes been compared to empirical data?
- Model epidemiology has been compared available literature reporting on the prevalence or time spent in various mood states (Kupka et al. 2007). Differences are

discussed in the Discussion section of the manuscript, following heterogeneity in the available literature.

Part E: Other validation techniques

**Other validation techniques:** Have other validation techniques been performed?
- The TECH-VER black box checklist.

Verification Checklist to Reduce Errors in Models and Improve Their Credibility (TECH-VER)

**Test description**	**Conclusions (*Expected result of the test in cursive*)**
Pre-analysis calculations	
Does the technology (drug/device, etc.) acquisition costs increase with higher prices?	Yes
Does the drug acquisition cost increase for higher weight or body surface area?	NA
Does the probability of an event, derived from an odds ratio (OR)/ relative risk (RR) / hazard ratio (HR) and baseline probability, increases with higher OR/RR/HR?	Yes
If survival parametric distributions are used in the extrapolations, can the formulae used for the Weibull (generalized gamma) distribution generate the values obtained from the exponential (the Weibull or Gamma) distribution(s) under some parameter transformations?	Yes
In a partitioned survival model, does the progression free survival curve or the time on treatment curve crosses the overall survival curve?	NA
If survival parametric distributions are used in the extrapolations or time-to-event calculations, can the formulae used for the Weibull (generalized gamma)	Yes

distribution generate the values obtained from the exponential (the Weibull or Gamma) distribution(s) after replacing/transforming some of the parameters?	
Is hazard ratio calculated from Cox proportional hazards model applied on top of the parametric distribution extrapolation found from the survival regression?	NA
For the treatment effect inputs, if the model uses outputs from WINBUGs, are the OR, HR and RR values all within plausible ranges? (should be all non-negative and the average of these WINBUGs outputs should give the mean treatment effect)	NA
Event-state calculations	
Calculate the sum of the number of patients at each health state	Adds up to the cohort size
Check if all probabilities and number of patients in a state are greater than or equal to zero	Yes
Check if all probabilities are smaller than or equal to one	Yes
Compare the number of dead (or any absorbing state) patients in a period with the number of dead (or any absorbing state) patients in the previous periods?	Larger as it should.
In case of lifetime horizon, check if all patients are dead at the end of the time horizon	Yes
Discrete event simulation specific: sample one of the "time to event" types used in the simulation from the specified distribution. Plot the samples and compare the mean and the variance from the sample	NA
Set all utilities to one<br><br>Set all utilities to zero	*The QALYs accumulated at a given time would be the same as the life years accumulated at that time* -> correct.<br><br>*No utilities will be accumulated in the model* -> correct.

Decrease all state utilities simultaneously (but keep event based utility decrements constant)	*Lower utilities will be accumulated each time* -> correct.
Set all costs to zero	*No costs will be accumulated in the model at any time* -> correct.
Put mortality rates to 0	*Patients never die*-> correct.
Put mortality rate extremely high	*Patients die in the first few cycles* -> correct.
Set the effectiveness, utility and safety related model inputs for all treatment options equal	*Same life years and QALYs should be accumulated for all treatment at any time* -> correct.
In addition to the inputs above, set cost related model inputs for all treatment options equal	*Same costs, life years and QALYs should be accumulated for all treatment at any time* -> correct.
Change around the effectiveness, utility and safety related model inputs between two treatment options	*Accumulated life years and QALYs in the model at any time should be also reversed* -> correct.
Check if the number of alive patients estimate at any cycle is in line with general population life table statistics	*At any given age, the % alive should be lower or equal in comparison to the general population estimate* -> correct.
Check if the QALY estimate at any cycle is in line with general population utility estimates	*At any given age, the utility assigned in the model should be lower or equal in comparison to the general population estimate* -> correct.
Set the inflation rate of the previous year higher	*The costs (which are based on a reference from previous years) assigned at each time will be higher* -> correct.
Calculate the sum of all ingoing and outgoing transition probabilities	*Both should be one* -> correct.

Calculate the number of patients entering and leaving a tunnel state throughout the time horizon	*Numbers entering = Numbers leaving* -> correct.
Check if the time conversions for probabilities were conducted correctly.	Yes
Decision tree specific: calculate the sum of the expected probabilities of the terminal nodes	NA
Patient-level model specific: check if common random numbers are maintained for sampling for the treatment arms?	Yes
Patient-level model specific: check if correlation in patient characteristics is taken into account when determining starting population?	Yes
Increase the treatment acquisition cost	*Costs accumulated at a given time will increase during the period when the treatment is administered* -> correct.
Population model specific: set the mortality and incidence rates to zero	*Prevalence should be constant in time* -> correct.
Result calculations	
Check the incremental life years and QALYs gained results. Are they in line with the comparative clinical effectiveness evidence of the treatments involved?	In the case study MBCT reduces the probability of having a depressive relapse, and increases the probability of a manic relapse. According to the model MBCT gains QALYs and Lys, which is expected as the QoL and probability of death are greater during depression.
Check the incremental cost results. Are they in line with the treatment costs?	Total incremental costs for MBCT are lower than for CAU when looking from a societal perspective (due to productivity losses), and higher when looking from a healthcare perspective. Treatment costs are higher because the probability of a manic episode is larger, which is more

	costly episode compared a depressive episode.
Total life years > total quality adjusted life years	Yes
Undiscounted results > discounted results	Yes
Divide undiscounted total QALYs by undiscounted life years.	*This value should be within the outer ranges (maximum and minimum) of the all utility value inputs*. -> Correct (0.7)
Subgroup analysis results: How do the outcomes change if the characteristics of the baseline change?	*Better outcomes for better baseline health conditions and worse outcomes for worse health conditions are expected.* -> correct. (utilities of remission vs. mania/depression.)
Could you generate all the results in the report from the model (including the uncertainty analysis results)?	Yes
Does the total life years, QALYs and costs decrease if a shorter time horizon is selected?	Yes
Is the reporting and contextualization of the incremental results correct?	*The use of the terms such as: "dominant"/ "dominated"/ "extendedly dominated"/ "cost-effective" etc. should be in line with the results.*<br><br>*In the incremental analysis table involving multiple treatments, ICERs should be calculated against the next non-dominated treatment.*
Are the reported ICERs in the fully incremental analysis non-decreasing?	Yes
If disentangled results are presented, do they sum up to the total results? (e.g. different cost types sum up to the total costs estimate)	NA

Check if half cycle correction is implemented correctly (total life years with half cycle correction should be lower than without)	Correct
Check the discounted value of costs/QALYs after 2 years	Correct
Set discount rates to zero	*The discounted and undiscounted results should be the same* -> correct.
Set mortality rate to zero	*The undiscounted total life years per patient should be equal to the length of the time horizon* -> correct.
Put the consequence of adverse event/discontinuation to zero. (zero costs and zero mortality/utility decrements)	NA.
Divide total undiscounted treatment acquisition costs by the average duration on treatment.	Total treatment acquisition costs = (cost per session/hour *no. of sessions*2.5 hours)/ session group size) =(142*8*2.5)/10=284
Set discount rates to a higher value	*Total discounted results should decrease* -> correct.
Set discount rates of costs/effects to an extremely high value	*Total discounted results should be more or less the same as the discounted results accrued in the first cycles* -> correct.
Put adverse event/discontinuation rates to zero and then to extremely high level.	NA
Double the difference in efficacy and safety between new intervention and comparator and report the incremental results.	*Approximately twice of the incremental effect results of the base case.*<br><br>Basecase: RR depression 1.32, RR mania 0.81 -> incremental QALY = 0.02<br><br>Test: RR depression 1.6, RR mania 0.6 -> incremental QALY = 0.04

Do the same for a scenario in which the difference in efficacy and safety is halved.	Basecase: RR depression 1.32, RR mania 0.81 -> incremental QALY = 0.02<br><br>Test: RR depression 1.15, RR mania 0.9 -> incremental QALY = 0.01
Uncertainty analysis calculations	
Are all parameters subject to uncertainty included in the one-way sensitivity analysis (OWSA)?<br><br>Check if the OWSA includes any parameters associated with joint uncertainty (e.g. parts of a utility regression equation, survival curves with multiple parameters).	Yes<br><br>No
Are the upper and lower bounds used in the one-way sensitivity analysis used confidence intervals based on the statistical distribution assumed for that parameter?<br><br>Are the resulting ICER, incremental costs/QALYs with upper and lower bound of a parameter plausible and in line with a priori expectations?	Yes, if possible. If no interval is available a 20% deviation is applied.<br><br>Yes
Check that all parameters used in the sensitivity analysis have an appropriate associated distributions<br><br>- upper and lower bounds should surround the deterministic value (i.e. Upper bound ≥ mean ≥ Lower bound)<br><br>- standard error and not standard deviation used in sampling<br><br>- Lognormal / gamma distribution for hazard ratios and costs/ resource use<br><br>- Beta for utilities and proportions/probabilities<br><br>- Dirichlet for multinomial<br><br>- Multivariate normal for correlated inputs (e.g. survival curve or regression parameters)<br><br>- Normal for other variables as long as samples don't violate requirement to remain positive when appropriate	Correct.

Check PSA output mean costs, QALYs and ICER compared to the deterministic results. Is there a large discrepancy?	No
If you take new PSA runs from the excel model do you get similar results?	Yes
Is(are) the CEAC line(s) in line with the CE scatter plots and the efficient frontier?	Yes
Does the PSA cloud demonstrate an unexpected behavior or has an unusual shape?	No
Is the sum of all CEAC lines equal to 1 for all WTP values?	Yes
Are the explored scenario analyses provide a balanced view on the structural uncertainty? (i.e. not always looking at more optimistic scenarios)	Yes
Are the scenario analysis results plausible and in line with a priori expectations?	Yes
Check the correlation between 2 PSA results (i.e. costs/QALYs under the SoC and costs/QALYs under the comparator)	Correct
If a certain seed is used for random number generation (or previously generated random numbers are used), check if they are they scattered evenly between 0-1 when they are plotted?	NA
Compare the mean of the parameter samples generated by the model against the point estimate for that parameter, use graphical methods to examine distributions, functions	NA
Check if sensitivity analyses include any parameters associated with methodological/ structural uncertainty (e.g. annual discount rates, time horizon).	No
Value of information analysis if applicable: Was this implemented correctly?	NA

Which types of analysis? Were aggregated parameters used? Which parameters are grouped together? Does it match the write-up's suggestions?<br><br>Is EVPI larger than all individual EVPPI?<br><br>Is EVPPI for a (group of) parameters larger than the EVSI of that (group) of parameter(s)?<br><br>Are the results from EVPPI in line with OWSA or other parameter importance analysis (e.g. ANCOVA)?	
Did the electronic model pass the black-box tests of the previous verification stages in all PSA iterations and in all scenario analysis settings? (additional macro can be embedded to PSA code, which stops the PSA when an error such as negative transition probability, is detected)	Yes
Check the correlation between 2 PSA results (i.e. costs/QALYs under the SoC and costs/QALYs under the comparator)	Correct
OWSA=one-way sensitivity analysis; ICER = incremental cost-effectiveness ratio; PSA = probabilistic sensitivity analysis; WTP = willingness to pay; CE = cost-effectiveness; CEAC = cost-effectiveness acceptability curve; LY = life years; QALYs = Quality adjusted life years; OR = odds ratio; RR= relative risk; HR = hazard ratio	
